# PSMD2-Mediated MAPK Signaling Promotes Bladder Cancer Development and Immune Microenvironment Remodeling

**DOI:** 10.32604/or.2025.072373

**Published:** 2026-03-23

**Authors:** Shuwen Sun, Jingcheng Zhang, Zongtai Zheng, Yajuan Hao, Tianyuan Xu, Ji Liu, Liang Sun, Aimin Wang, Yadong Guo, Shiyu Mao, Xu Zhang, Yunfei Xu, Yifan Chen, Yang Yan

**Affiliations:** 1 Department of Urology, Shanghai Tenth People’s Hospital, School of Medicine, Tongji University, Shanghai, 200072, China; 2 Department of Urology, Bengbu First People’s Hospital, Bengbu, 233000, China; 3 Urologic Cancer Institute, School of Medicine, Tongji University, Shanghai, 200072, China; 4 Department of Nuclear Medicine, Shanghai Tenth People’s Hospital, School of Medicine, Tongji University, Shanghai, 200072, China; 5 Department of Urology, The Affiliated Guangdong Second Provincial General Hospital of Jinan University, Guangzhou, 510317, China; 6 Bio-X Institutes (Key Laboratory for the Genetics of Development and Neuropsychiatric Disorders), Shanghai Jiao Tong University, Shanghai, 200240, China

**Keywords:** Bladder cancer (BCa), proteasome 26S subunit non-ATPase 2 (PSMD2), C-X-C motif chemokine ligand 14 (CXCL14), immune infiltration

## Abstract

**Objectives:**

Bladder cancer (BCa) progression is closely linked to the immune microenvironment. However, the key molecules that regulate this microenvironment and their specific mechanisms remain poorly understood. This study aims to identify a key molecule and elucidate its mechanisms, providing a theoretical basis for identifying novel therapeutic targets.

**Methods:**

Immune microenvironment-related genes in BCa were identified using The Cancer Genome Atlas and Shanghai Tenth People’s Hospital datasets. Proteasome 26S subunit non-ATPase 2 (PSMD2) expression was validated via quantitative polymerase chain reaction (qPCR), Western blot (WB) analysis, and immunofluorescence (IF). *In vitro* and *in vivo* experiments confirmed the role of PSMD2 in cell proliferation, invasion, and migration. Kyoto encyclopedia of genes and genomes (KEGG) and Gene Ontology (GO) analyses were conducted to assess PSMD2’s influence on immune microenvironment remodeling. A pathomics model predicted PSMD2 expression in patients with BCa.

**Results:**

PSMD2 was identified as a critical factor in BCa, with high expression correlating with poor prognosis and tumor progression. Mechanistically, PSMD2 enhances malignancy by promoting mitogen-activated protein kinase kinase (MEK) and extracellular signal-regulated kinase (ERK) phosphorylation within the mitogen-activated protein kinase (MAPK) signaling pathway. Combined bioinformatics and experimental analyses reveal that PSMD2 downregulates chemokine (C-X-C motif) ligand 14 (CXCL14) expression and secretion via the MAPK pathway, thereby remodeling the immune microenvironment and driving tumor progression. Pathomics analysis further supports the potential of PSMD2 expression as a predictive marker in BCa tissues.

**Conclusion:**

PSMD2 is overexpressed in BCa and significantly correlates with poor prognosis and tumor progression. It promotes malignant development and immune microenvironment remodeling through the MAPK pathway. Pathological analysis can predict PSMD2 expression, offering valuable insights into immunotherapy responses and survival outcomes.

## 1 Introduction

Bladder cancer (BCa), a common malignancy of the urinary system, results in approximately 613,791 new cases and 220,349 deaths annually [1]. The age-standardized incidence of bladder cancer is characterized by regional disparities and a projected persistent rise [2]. Approximately 75% of BCa cases are non-muscle-invasive (NMIBC) [3], with the remaining 25% classified as muscle-invasive BCa (MIBC), where the cancer has infiltrated deeper layers. Patients with MIBC experience significantly lower five-year survival rates compared to those with NMIBC and often require multimodal treatments such as chemoradiotherapy and immunotherapy. Despite rapid advancements in cancer immunotherapy, a substantial proportion of patients with advanced BCa do not respond to immunotherapy, losing a critical treatment option [4]. Therefore, identifying factors that influence BCa progression and the immune microenvironment is crucial for improving immunotherapy efficacy and extending patient survival.

Proteasome 26S subunit non-ATPase 2 (PSMD2) is a key component of the 19S regulatory particle. As a non-ATPase subunit, it contributes to the proteasomal degradation process [5]. Extensive studies have demonstrated that PSMD2 plays a key role in the progression of breast cancer [6], liver cancer [7], and lung adenocarcinoma [8]. Additionally, elevated PSMD2 expression is associated with poorer overall and disease-free survival rates in BCa, marking it as an important prognostic biomarker in BCa [9]. However, the precise mechanisms underlying PSMD2’s role in BCa and its impact on the immune microenvironment remain unclear.

The tumor microenvironment (TME) is enriched with various chemokines [10], which primarily regulate immune cell movement and positioning within tissues. These molecules attract specific immune cell subsets into the tumor and reshape the TME, influencing tumorigenesis and metastasis. Chemokine-receptor interactions facilitate the recruitment of distinct immune cell types into the TME [11], with each subset exerting differential effects on tumor progression and therapeutic response. For example, in melanoma, a lack of C-C chemokine receptor type 5 (CCR5) ligands (C-C motif chemokine ligand 3 (CCL3), C-C motif chemokine ligand 4 (CCL4), C-C motif chemokine ligand 5 (CCL5)) and C-X-C chemokine receptor type 3 (CXCR3) ligands (C-X-C motif chemokine ligand 9 (CXCL9), C-X-C motif chemokine ligand 10 (CXCL10)) is linked to a reduced presence of antigen-specific T cells [12]. In contrast, in breast, colorectal, lung, ovarian cancers, and melanoma, elevated levels of CXCL9, CXCL10, and C-X-C motif chemokine ligand 11 (CXCL11) correlate with an increased presence of tumor-infiltrating T cells and natural killer (NK) cells [13,14]. Additionally, previous studies have shown that CXCL14 attracts NK cells, activates monocytes at sites of inflammation or malignancy, enhances the recruitment of surrounding immune cells, and induces apoptosis [15].

The clinical diagnosis of BCa predominantly relies on histopathological examination, where pathologists analyze tissue samples through medical microscopy and direct visual assessment. However, certain histopathological patterns may present ambiguity, leading to potential misinterpretation [16]. Traditional immunohistochemical techniques are often inadequate for complex differential diagnoses. Automated pathology imaging systems offer a promising and effective alternative. By leveraging high-throughput medical image processing, these systems extract valuable high-dimensional data, playing a pivotal role in advancing precision medicine [17,18]. These systems are increasingly utilized to analyze medical images in various cancers, including breast cancer [19], lung adenocarcinoma [20], and skin cancer [21]. A study by Chen et al. further supports the efficacy of machine learning-derived pathomic features in diagnosing BCa and predicting patient survival [22].

This study aims to systematically validate the critical role of PSMD2 in the development and progression of bladder cancer by integrating database mining and fundamental experiments. Additionally, a pathological analysis model will be constructed to predict its expression levels, thereby evaluating patients’ sensitivity to immunotherapy and their survival prognosis. Furthermore, this research will screen and identify potential lead compounds targeting PSMD2, laying the foundation for subsequent experimental validation and drug development.

## 2 Materials and Methods

### 2.1 Data Acquisition

TCGA (https://www.cancer.gov/ccg/research/genome-sequencing/tcga) and Gene Expression Omnibus (https://www.ncbi.nlm.nih.gov/GEO/) and BCa RNA sequencing data (HRA009938, https://ngdc.cncb.ac.cn/gsa-human) from Shanghai Tenth People’s Hospital (STPH) were used in this study. The total RNA extraction, bilateral library generation, RNA sequencing protocol, patient inclusion and exclusion criteria included in STPH’s BCa sequencing library establishment process have been described in our published article [23]. The study was conducted according to the guidelines of the Declaration of Helsinki, and approved by the Shanghai Tenth People’s Hospital Ethics Committee (Approval No. 2021KN108 and No. 24KT68) and Guangdong Second Provincial General Hospital Ethics Committee (Approval No. 2024-KY-KZ-128-01), and informed consent was collected from all participants. The animal experiments were approved by the Shanghai Tenth People’s Hospital Ethics Committee (Approval No. SHDSYY-2024-1533). At the same times, the best databases [24] were also used in this study.

### 2.2 Cell Lines and Cell Culture

T24 (CBP60649), 5637 (CBP60309), UMUC3 (CBP60753), J82 (CBP60311), and RT4 (CBP60313) (All cell lines were sourced from the Chinese Academy of Sciences, Shanghai, China) were used as human BCa cell lines and MB49 (CBP61301, Chinese Academy of Sciences) was used as mouse BCa cell line in this study. Immortalized human normal bladder epithelial cell line SV-HUC-1 (CBP61685, Chinese Academy of Sciences) was also used in this study. MB49, T24, 5637, and UMUC3 cell lines were cultured in RPMI-1640 medium (Thermo Fisher Science, 11875085, Waltham, MA, USA). J82 was cultured in DMEM medium (Thermo Fisher Science, 11965092). RT4 cell lines were cultured in McCoy’s 5A medium (Thermo Fisher Science, 16600082). SV-HUC-1 was cultured in F12k medium (Sigma Aldrich, N3520, St. Louis, MO, USA). The media used in the culture process were all supplemented with a final concentration of 1% penicillin/streptococcus (Hyclone, SV30082.01, Logan, UT, USA) and 10% fetal bovine serum (FBS; Thermo Fisher Science, 16000044). These cell lines were cultured at 37°C in an incubator (Thermo Fisher Science, 311) at 5% CO_2_. All cells have undergone mycoplasma testing and cell line authentication, with detailed results provided in the supplementary materials.

### 2.3 CD8^+^ T Cell Isolation, Sorting, Activation

Cord blood and PBMC of healthy donors were isolated by density gradient centrifugation (Cell pellets were resuspended in equal-volume normal saline and carefully layered over Ficoll-Paque (MERCK, GE17-1440-02, Hesse, Germany). Following centrifugation at 1000× *g* for 20 min at 20°C with the brake off, the middle white lymphocyte ring layer was harvested into a fresh tube, washed with normal saline, and centrifuged at 450× *g* for 5 min. After discarding the supernatant, red blood cells were lysed with RBC lysis buffer (Thermo Fisher Science, 00-4333-57) for 10 min at room temperature (RT) in the dark. Lysis was terminated with normal saline, followed by centrifugation at 450× *g* for 5 min at RT. The supernatant was discarded to obtain isolated lymphocytes. CD8^+^ T cells were stained with anti-CD8 (1 μg/mL; BioLegend, 980908, San Diego, CA, USA) and CD3 (1 μg/mL; BioLegend, 981012) antibodies and purified by FACS Aria III (BD Biosciences, BD FACSAria™ III, San Jose, CA, USA). On day 3 after stimulation with CD3/28 antibodies (1 μg/mL; Thermo Fisher Science, 11161D), the culture was supplemented every other day with IL-7 (5 ng/mL; PrimeGene Technology, GMP-101-07, Beijing, China) and IL-15 (5 ng/mL; PrimeGene Technology, GMP-101-15) in complete X-VIVO (5% FBS; Thermo Fisher Science). Continued expansion for *in vitro* experiments.

### 2.4 Western Blot (WB)

RIPA (Beyotime, P0013B, Shanghai, China) is utilized to completely disrupt cells or tissues for a duration of 30 min. After this, the samples are centrifuged at 13500× *g* at 4°C for 10 min. Supernatant is collected and the protein concentration is determined using the Bicinchoninic Acid (BCA) protein assay (Beyotime, P0010). For Western blot analysis, proteins are quantified to a concentration of 80 μg and subjected to electrophoresis on a 10% sodium dodecyl sulfate-polyacrylamide gel (Beyotime, P0690). The proteins are then transferred onto nitrocellulose membrane (MERCK, HATF00010). The membranes are pre-incubated with a solution of skim milk in PBST containing 5% at RT for 1 h to prevent non-specific binding.

Following the blocking step, the membranes are incubated with the diluted primary antibodies at 4°C for a period exceeding 12 h. Post-incubation, the membranes are washed three times with PBST and incubated with a horseradish peroxidase (HRP)-conjugated secondary antibody for 1 h at RT. The proteins are detected using a chemiluminescent imaging system (Tanon 5200 System, Shanghai, China), and their concentrations are quantified accordingly.

This study used the following primary antibodies: β-actin, 1:5000 dilution (ProteinTech, 66009-1-Ig, Wuhan, China), PSMD2, 1:1000 dilution (ProteinTech, 14748-1-AP), CXCL14, 1:1000 dilution (Abmart, TD12377, Shanghai, China), ERK1/2, 1:1000 dilution (Affinity, AF0155, Cincinnati, USA), MEK1/2, 1:1000 dilution (Affinity, AF6385), p-ERK1/2, 1:1000 dilution (Affinity, AF1015), and p-MEK1/2, 1:1000 dilution (Affinity, AF8035). The secondary antibody: HRP-Rabbit, 1:10000 dilution (Abcam, ab6721, Cambridge, UK), HRP-Mouse, 1:10000 dilution (Abcam, ab6728)

### 2.5 RNA Isolation and Quantitative RealTime Quantitative Polymerase Chain Reaction PCR (qPCR)

Total RNA is isolated from human samples, either 300 mg tissue or 3 × 10^6^ cells, employing the RNA Extraction Kit (Servicebio, G3689, Wuhan, China). Subsequently, the first strand of cDNA is synthesized utilizing the Reverse Transcription System Kit (Vazyme, R323, Nanjing, China). The 20 μL reaction mixture contained 1 μg of total RNA, and the reaction was carried out under the following conditions: 37°C for 15 min, then 85°C for 5 s. Quantitative PCR (qPCR) is conducted with the ABI Prism 7500 Sequence Detection System (Applied Biosystems, CA, USA) and ChamQ Universal SYBR qPCR Master Mix (Vazyme, Q711, Nanjing, China).

The qPCR parameter was set as follows: 95°C for 5 min, followed by 40 cycles consisting of 95°C for 10 s and 60°C for 30 s. β-actin serves as the endogenous reference gene. The relative quantification of mRNA expression is determined by applying the 2^−ΔΔCT^ method.

The forward and reverse primers for each target gene are as follows: β-actin, F: AGAGCCTCGCCTTTGCC, R: GGGGTACTTCAGGGTGAGGA; PSMD2, F: TGCTCGTGGAACGACTAGG, R: CAGTTTGCCATAGTGTGGACG; CXCL14, F: CGCTACAGCGACGTGAAGAA, R: GTTCCAGGCGTTGTACCAC; GLI1, F: AGCGTGAGCCTGAATCTGTG, R: CAGCATGTACTGGGCTTTGAA; STON2, F: ACCATGTGATTGCCACCCAC, R: AGCTCTCGGACTGGTCTGG; SCN4B, F: GGCTTTTGGTGGAAGAAGTGG, R: AAGACTGTGGGGGATCTGGT; POLR1C, F: CGCAATGTCCATACTACTGACTT, R: CCACACGGAAATTCTTCTCGAA; CXCL8, F: GAAGTTTTTGAAGAGGGCTGAGA, R: ACCAAGGCACAGTGGAACAA; CCL18, F: ATTCTGAAACCAGCCCCCAG, R: GGGCATAGCAGATGGGACTC; CCL14, F: CCAAGCCCGGAATTGTCTTCA, R: GGGTTGGTACAGACGGAATGG; CCL16, F: CTTATCATTACTTCGGCTTCTCGC, R: GGCCTTTCTGTATCCCACCACTA.

### 2.6 Lentivirus Infection and Cell Transfection

The 10 μg plasmid was cloned into a lentiviral vector. HEK-293T cells, cultured in serum-free DMEM in a 10 cm dish, were then transfected using 10 μL of the specified reagent (Genomeditech, GMeasy-10, Shanghai, China). Subsequently, 18 h post-transfection, the medium was replaced with fresh complete DMEM containing serum. Supernatants were collected at 24 and 48 h after the medium change and stored at −80°C. For transfection, 1 × 10^5^ 5637, T24, and MB49 cells were seeded per well in a 6-well plate. Once the cells reached approximately 80% confluence, the lentivirus at a multiplicity of infection of 5 was added for 48 h, and the cells underwent 4 μg/mL puromycin-based drug selection for two weeks. Transfection success was confirmed via qPCR and WB analysis. (Sequence details are presented in Supplementary Information, Table S1).

### 2.7 5-Ethynyl-2^′^-deoxyuridine (EdU) Incorporation Assay

To assess cell proliferation, 4 × 10^3^ cells were seeded per well in 96-well plates. After 24 h, the EdU assay kit (APExBIO Corporation, K1075, Houston, TX, USA) was used to evaluate proliferation. The procedure was briefly as follows: Cells were incubated with 10 μM EdU solution in a cell culture incubator for 2 h. Subsequently, they were fixed, permeabilized, and washed three times with PBS. Following this, a Click reaction mixture was added, and the cells were incubated at RTin the dark for 30 min. After another PBS wash, nuclei were counterstained with Hoechst. Fluorescent images were captured with a fluorescence microscope (Nikon, TS2R-FL, Tokyo, Japan). Cell counting was performed using ImageJ software (version 1.53K).

### 2.8 Migration and Invasion Assays

Invasive and migratory capabilities of BCa cells were evaluated using transwell inserts (Yeasen, 84051ES, Shanghai, China), for the invasion assay, the inserts were pre-coated with 100 μL of Matrigel (1.0 mg/mL) (Yeasen, 40183ES, Shanghai, China). The insert was placed in a 24-well plate, and 2.5 × 10^4^ cells were seeded in the upper chamber with an FBS-free medium. In the lower chamber, 600 μL of medium containing 10% FBS was added. After a 24-h incubation at 37°C, the insert was washed with PBS, fixed with 4% paraformaldehyde, and cells on the upper surface were removed with a cotton swab. The cells were then stained with 0.5% crystal violet (Yeasen, 60506ES, Shanghai, China) at RTfor 5 min, rinsed with PBS, air-dried, and documented using a microscope (Nikon, TS2R-FL). For evaluating the migratory capabilities of CD8^+^ T cells, 5 × 10^6^ CD8^+^ T cells were seeded in the upper compartment of transwell inserts (Yeasen, Shanghai, China) with 600 μL FBS-free medium, while 200 μL of different tumor cell supernatants were added to the lower chamber. After 24 h of incubation at 37°C, the lower compartment was gently washed with PBS, and the mixture was centrifuged at 600× *g* for 5 min under RTconditions. The supernatant was removed, and the cells were stained with 0.5% crystal violet for 20 min. The results were documented using a microscope (Nikon, TS2R-FL). Cell counting was performed using ImageJ software (version 1.53K).

### 2.9 Flow Cytometry

Human CD8^+^ T cells were subjected to phenotype analysis by flow cytometry using Abs specific to human CD3 (1 μg/mL; BioLegend, 981012), CD8 (1 μg/mL; BioLegend, 980908). Mouse CD8^+^ T cells and NK cells were subjected to phenotype analysis by flow cytometry (BD Biosciences, San Jose, CA, USA) using Abs specific to mouse CD45 (1 μg/mL, BioLegend, S18009F), CD8 (1 μg/mL, BioLegend, 53-5.8), NK1.1 (1 μg/mL, BioLegend, S17016D) and CXCL14 were subjected to phenotype analysis by flow cytometry using Abs specific to human CXCL14 (1 μg/mL; Abcam, EPR22807-28). The data were analyzed using FlowJo software (Flow V10.8.1, BD Life Sciences). All flow cytometry staining was performed at 4°C, protected from light, for 40–60 min, with >10,000 singlet events acquired per sample.

### 2.10 Image Annotation and Preparation

To train a deep learning model for distinguishing between tumor and non-tumor regions in BCa hematoxylin and eosin (H&E) stained whole-slide images (H&E WSIs), 70 WSIs from the TCGA dataset were randomly chosen for model training, while 30 were allocated for internal validation. For external validation, 10 WSIs each were selected from the STPH and Guangdong Second Provincial General Hospital (GD2H) datasets. Tumor areas were delineated by a pathologist with extensive experience in BCa using QuPath software (version 0.3.2, Queen’s University Belfast). These WSIs were then divided into 224 × 224 pixel patches at 20× magnification (112 μm × 112 μm) using the Python package Openslide (version 4.0.0), with patches labeled as either tumor or normal tissue. Prior to deep learning model training, edge detection was employed via OpenCV (version 4.10.0, threshold set at 0.02) to exclude patches that were primarily blank. Color normalization was then performed on all patches using the Reinhard method in OpenCV to ensure uniform color representation across datasets.

To classify tumor and normal patches, transfer learning was implemented using the ResNet50 model in PyTorch (version 2.4.1). The model training ran for 50 epochs with stochastic gradient descent (SGD), an initial learning rate of 0.1, momentum of 0.9, and weight decay set at 0.001. Cross-entropy loss was the chosen criterion, and model performance was assessed on both an internal validation set and two external validation sets from STPH and GD2H. The model performance was evaluated using accuracy, sensitivity, specificity, positive predictive value (PPV), negative predictive value (NPV), and the receiver operating characteristic (ROC).

### 2.11 Other Materials and Methods

Patient information (patient characteristics are outlined in supplementary information Table S2), details of model construction and evaluation, subcutaneous xenograft model, immunohistochemistry (IHC) and immunofluorescence (IF) (the details of the antibodies are provided in Table S3), cell counting kit-8 assay, colony formation assay, wound healing assay, co-immunoprecipitation (Co-IP) assay, *in silico* virtual screening and molecular docking and statistical analysis can be found in the supplementary materials.

### 2.12 Data analysis

Quantitative data are presented as mean values ± SEM from at least three independent experiments. All statistical analyses were conducted using GraphPad Prism software (v8.0.2, GraphPad Inc, San Diego, CA, USA). Intergroup differences were evaluated using appropriate statistical tests: two-group comparisons employed unpaired Student’s *t*-tests, while multiple group comparisons utilized one way ANOVA followed by Tukey’s post-hoc test. A probability value of *p* < 0.05 was considered statistically significant for all analyses.

## 3 Results

### 3.1 The Relationship between PSMD2 and the Progression and Prognosis of BCa

**Figure 1:**
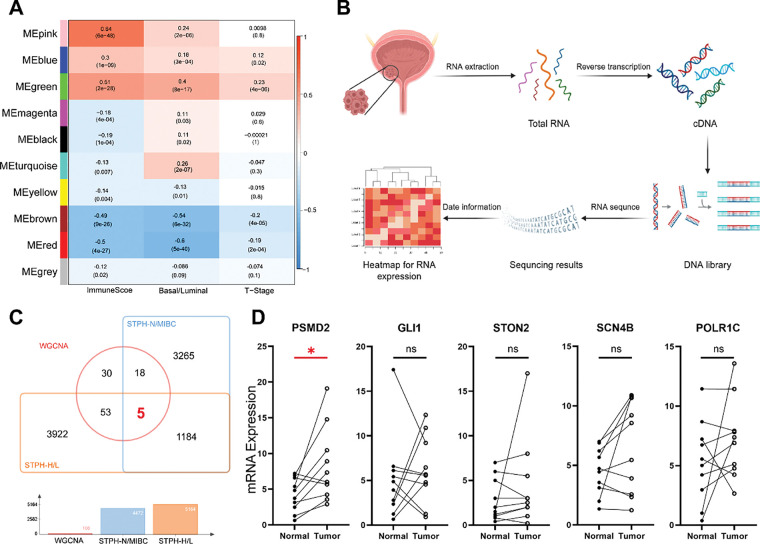

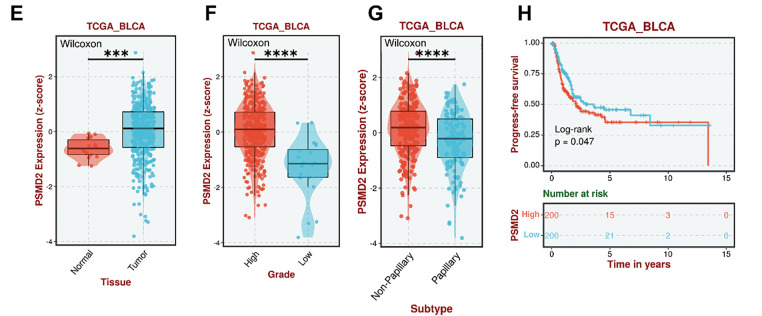
Relationship between proteasome 26S subunit non-ATPase 2 (PSMD2) and progression and prognosis of BCa. (**A**): Weighted gene co-expression network analysis (WGCNA) analysis of genetic modules most strongly associated with immune score, molecular type, and T stage in bladder cancer (BCa). (**B**): Flowchart of RNA-sequencing of BCa. (**C**): Venn diagrams revealed that the genes in the MEgreen gene module were highly expressed in both Shanghai Tenth People’s Hospital Muscle-Invasive Bladder Cancer (STPH-MIBC)-overexpressed and pathologically-graded tissues. (**D**): Quantitative polymerase chain reaction (qPCR) detection showing POLR1C, GLI1, STON2, SCN4B and PSMD2 expression in BCa and normal tissues. * *p* < 0.05, ns, not significant. (**E**–**H**): Gene expression of PSMD2 in tumor and normal tissues (**E**) and its correlation with grade. (**F**), subtype (**G**) and progress-free survival of BCa (**H**) in the cancer genome atlas (TCGA). ***p < 0.001, ****p < 0.0001

To investigate the genetic factors driving BCa progression, Weighted Gene Co-expression Network Analysis (WGCNA) was applied to the TCGA BCa dataset to identify gene modules most strongly associated with immune scores (derived using the ssGSEA algorithm [25,26]), molecular subtypes, and T stage. The analysis identified the MEgreen module as having the highest malignancy and strongest correlation (Fig. 1A). Tumor samples from patients with BCa at STPH were subsequently collected for RNA-seq analysis (Fig. 1B). By intersecting the 106 genes in the MEgreen module with upregulated genes in MIBC and high pathological grades from STPH, the following genes were identified: POLR1C, GLI1, STON2, SCN4B, and PSMD2 (Fig. 1C). qPCR validation of these five genes was performed using cancerous and adjacent normal tissues from 10 patients with BCa, revealing that only PSMD2 showed a statistically significant difference between the samples (Fig. 1D). Further analysis of the TCGA database confirmed that PSMD2 is highly expressed in BCa tissues (Fig. 1E), with expression levels increasing alongside pathological grade (Fig. 1F). Moreover, PSMD2 expression was significantly higher in non-papillary BCa (Fig. 1G) and correlated with progression-free survival (PFS) in patients (Fig. 1H).

To further validate these findings, several additional databases were analyzed. Consistent with the TCGA results, PSMD2 expression in BCa showed significant associations with patient age (Supplementary Fig. S1A), disease progression (Supplementary Fig. S1B), tumor grade (Supplementary Fig. S1C), cancer stage (Supplementary Fig. S1D), T category (Supplementary Fig. S1E), molecular subtypes (Supplementary Fig. S1F), response to platinum-based chemotherapy (Supplementary Fig. S1G), and survival period (Supplementary Fig. S1H).

### 3.2 PSMD2 is Highly Expressed in BCa Tissues and Cells

**Figure 2:**
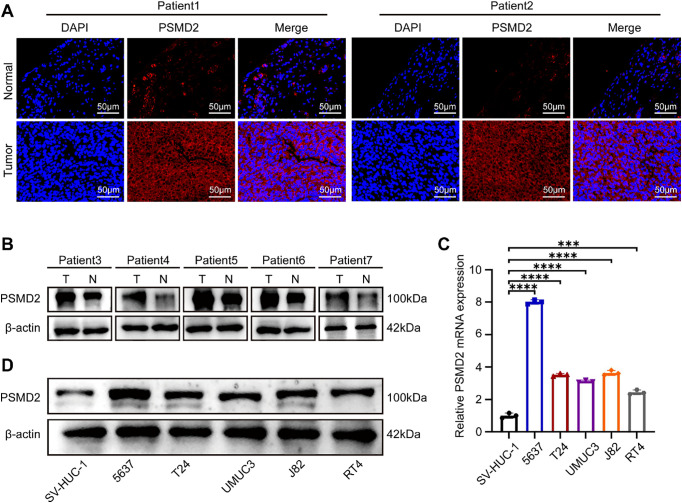
BCa tissues and cells highly express PSMD2. (**A**): IF images detailing the PSMD2 expression in the tumor and normal tissues (n = 2). Scale bars: 50 μm. (**B**): WB describing the PSMD2 expression in tumor and normal tissues (n = 5). (**C**): qPCR analysis displaying relative PSMD2 mRNA expression in BCa cell lines (n = 3). ***p < 0.001, ****p < 0.0001. (**D**): WB illustrating PSMD2 expression in BCa cell lines

Immunofluorescence (IF) analysis of PSMD2 expression in tissues revealed significantly elevated levels in cancerous tissues compared to adjacent normal tissues (Fig. 2A). Subsequently, PSMD2 protein levels were measured in cancerous and adjacent tissues from five patients, with WB analysis confirming that PSMD2 was markedly overexpressed in the cancerous tissues (Fig. 2B). PSMD2 expression was also examined in BCa cell lines. Using the SV-HUC-1 cell line (normal bladder cells) as a control, significantly upregulated PSMD2 mRNA levels were detected in five BCa cell lines (Fig. 2C). WB analysis further validated these findings, showing consistent overexpression of PSMD2 at the protein level (Fig. 2D). These experimental results demonstrate that PSMD2 is highly expressed in BCa tissues and cells.

### 3.3 PSMD2 Promotes the Proliferation of BCa Cells In Vivo

**Figure 3:**
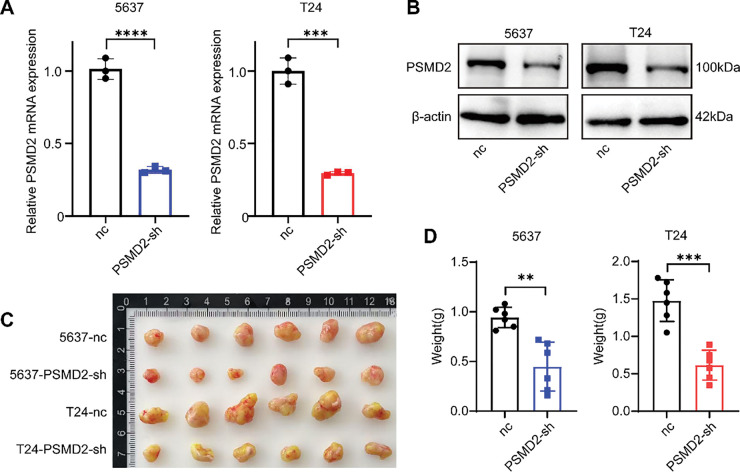

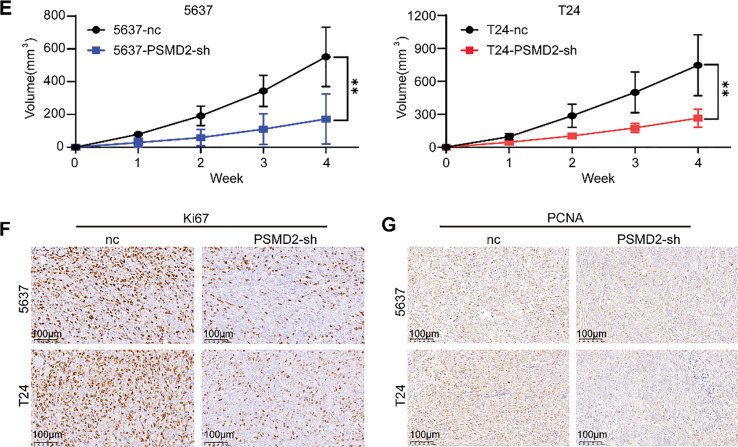
PSMD2 promotes the proliferation of BCa cells *in vivo*. (**A**,**B**): qPCR and western blot (WB) detection of knockdown of PSMD in 5637 and T24 cell lines. ***p < 0.001, ****p < 0.0001. (**C**): Image of tumor size in nude mice after subcutaneous tumor loading for four weeks. (**D**): Histogram depicting the difference in tumor weight between the nc group and PSMD2-sh group. n = 6. **p < 0.01, ***p < 0.001. (**E**): Tumor volume growth curve. **p < 0.01. (**F**,**G**): Representative immunohistochemistry (IHC) images of the antigen identified by monoclonal antibody Ki-67 (Ki67) (**F**) and proliferating cell nuclear antigen (PCNA) (**G**) in mouse subcutaneous tumor tissue. Scale bars: 100 μm

To investigate the role of PSMD2, two BCa cell lines, 5637 and T24, were selected for study. Stable PSMD2 knockdown cell lines were generated, and qPCR analysis confirmed a significant reduction in PSMD2 mRNA expression in both cell lines compared to the control group (Fig. 3A). WB analysis further verified the successful knockdown of PSMD2 in 5637 and T24 cells (Fig. 3B). To assess the *in vivo* effects of PSMD2, tumor-bearing experiments were conducted using nude mice. Significant differences in tumor volume, weight (Fig. 3C,D), and growth rate (Fig. 3E) were observed between the PSMD2 knockdown and control groups in both cell lines. The PSMD2 knockdown group exhibited reduced tumor growth and lower tumor weight. Immunohistochemical analysis of tumors from the mice showed decreased expression of Ki67 (Fig. 3F) and PCNA (Fig. 3G), which are markers of cell proliferation. These findings collectively support the conclusion that PSMD2 plays a critical role in promoting tumor proliferation *in vivo*.

### 3.4 Knockdown of PSMD2 Inhibits Bladder Cancer Progression In Vitro

**Figure 4:**
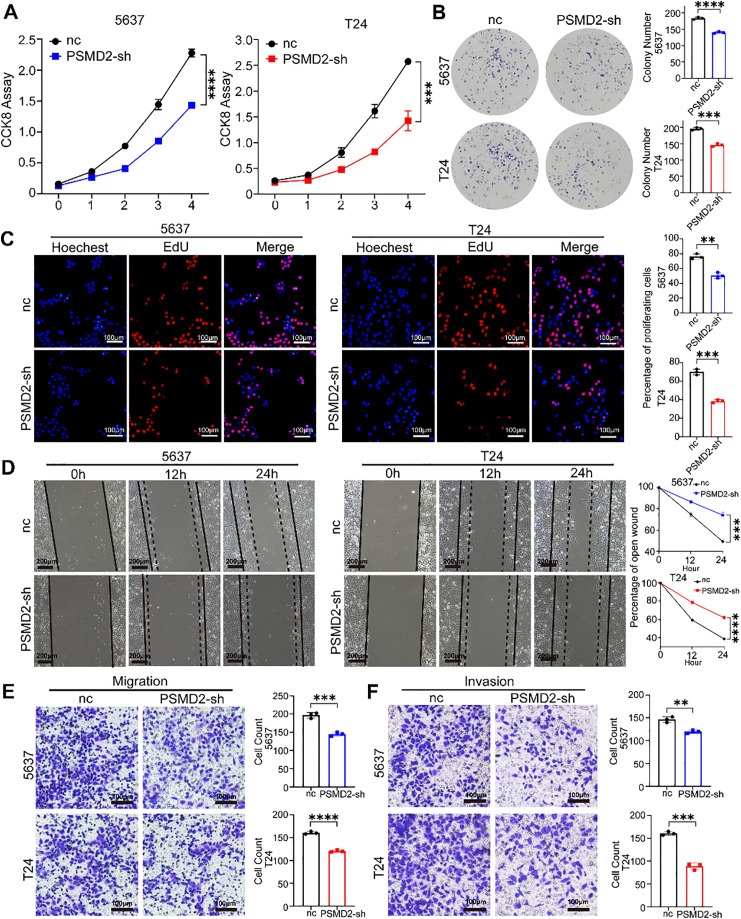
Knockdown of PSMD2 inhibits bladder cancer progression *in vitro*. (**A**–**C**): Cell counting kit-8 (CCK-8) experiment (**A**), colony formation assays (**B**) and EdU assay (**C**) were utilized to assess the viability of the 5637 and T24 cell lines following PSMD2 knockdown, respectively. **p < 0.01, ***p < 0.001, ****p < 0.0001. Scale bars: 100 μm. (**D**): Wound-healing assay was conducted to analyze the migration abilities of 5637 and T24 cell lines following PSMD2 knockdown, respectively. ***p < 0.001, ****p < 0.0001. Scale bars: 200 μm. (**E**,**F**): Transwell assay was executed to analyze the migratory (**E**) and invasive (**F**) capabilities of 5637 and T24 cell lines following PSMD2 knockdown, respectively. **p < 0.01, ***p < 0.001, ****p < 0.0001. Scale bars: 100 μm

To further explore the function of PSMD2 in BCa cells, a series of *in vitro* experiments were conducted. The CCK8 assay revealed a significant reduction in the proliferative capacity of the PSMD2-sh group compared to the negative control group (Fig. 4A). Plate cloning and EdU assays were also performed to assess cell proliferation. Consistent with the CCK8 results, both assays showed a decreased number of clones (Fig. 4B) and reduced proliferation (Fig. 4C) in the PSMD2-sh group. Furthermore, scratch and transwell assays were employed to evaluate the migratory and invasive abilities of T24 and 5637 cells. PSMD2 knockdown significantly diminished both migration (Fig. 4D,E) and invasion (Fig. 4F) in these cells. These results demonstrate that PSMD2 knockdown effectively inhibits the proliferation, migration, and invasion of BCa cells.

### 3.5 High PSMD2 Expression is Negatively Correlated with Immune Infiltration in Bladder Cancer

**Figure 5:**
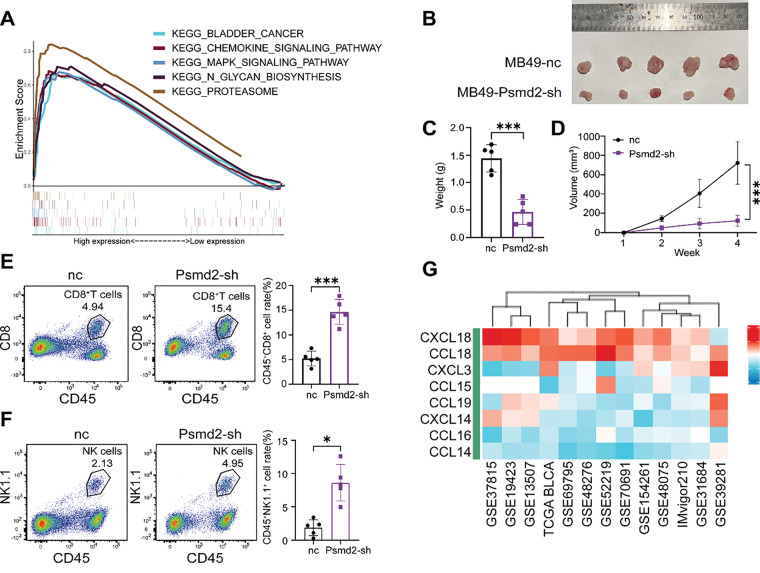

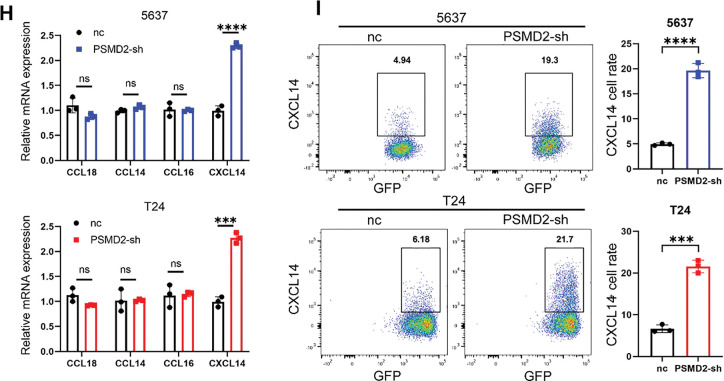
High PSMD2 Expression Is Negatively Correlated with Immune Infiltration in Bladder Cancer. (**A**): Analysis of pathways influenced by PSMD2 through KEGG enrichment. (**B**): Image of tumor size in C57 mice after subcutaneous tumor loading for four weeks. (**C**): Histogram depicting the difference in tumor weight between the nc group and Psmd2-sh group (n = 5). ***p < 0.001. (**D**): Tumor volume growth curve. ***p < 0.001. (**E**): Flow cytometry analysis the difference of the expression of CD8^+^ T cells between the nc group and Psmd2-sh group (n = 5). ***p < 0.001. (**F**): Flow cytometry analysis the difference of the expression of NK1.1^+^ cells between the nc group and Psmd2-sh group (n = 5). *p < 0.05. (**G**): Analysis of chemokines affected by PSMD2. (**H**): qPCR analysis displaying induced expression of CCL18, CCL14, CCL16, CXCL14 mRNA after knockdown of PSMD2 in 5637 and T24 cell line (n = 3). ***p < 0.001, ****p < 0.0001, ns no significance. (**I**): Flow cytometry analysis expression of CXCL14 after knockdown of PSMD2 in 5637 and T24 cell line (n = 3). ***p < 0.001, ****p < 0.0001

To explore how PSMD2 promotes BCa progression, KEGG enrichment analysis was performed using RNA-seq data from the STPH cohort. The results revealed significant enrichment in BCa-related pathways, including chemokine signaling, MAPK signaling, and CD8^+^ T cell activity, with PSMD2 expression closely associated with immune infiltration levels (Fig. 5A, Supplementary Fig. S2A,B). To further assess the impact of PSMD2 on the tumor immune microenvironment *in vivo*, subcutaneous tumor models were established in immunocompetent C57BL/6 mice. The data showed that both tumor growth rate and final tumor weight were significantly higher in the negative control (nc) group compared to the Psmd2 knockdown (Psmd2-sh) group (Fig. 5B–D). Flow cytometry analysis revealed increased infiltration of CD45^+^CD8^+^ T cells and CD45^+^NK1.1^+^ NK cells in Psmd2-sh tumors (Fig. 5E,F), indicating a negative correlation between Psmd2 expression and immune cell infiltration. Given the critical roles of chemokines and immune cells in tumor progression, this study screened for chemokines potentially regulated by PSMD2, identifying CCL18, CCL14, CCL16, and CXCL14 as potential downstream targets (Fig. 5G). qPCR validation confirmed that only CXCL14 was significantly upregulated upon PSMD2 knockdown (Fig. 5H). Subsequent flow cytometry analysis revealed increased CXCL14 protein levels in PSMD2-knockdown BCa cell lines (Fig. 5I), suggesting that PSMD2 may transcriptionally suppress CXCL14 expression.

### 3.6 PSMD2 Suppresses Chemokine CXCL14 Expression via the MAPK Pathway

**Figure 6:**
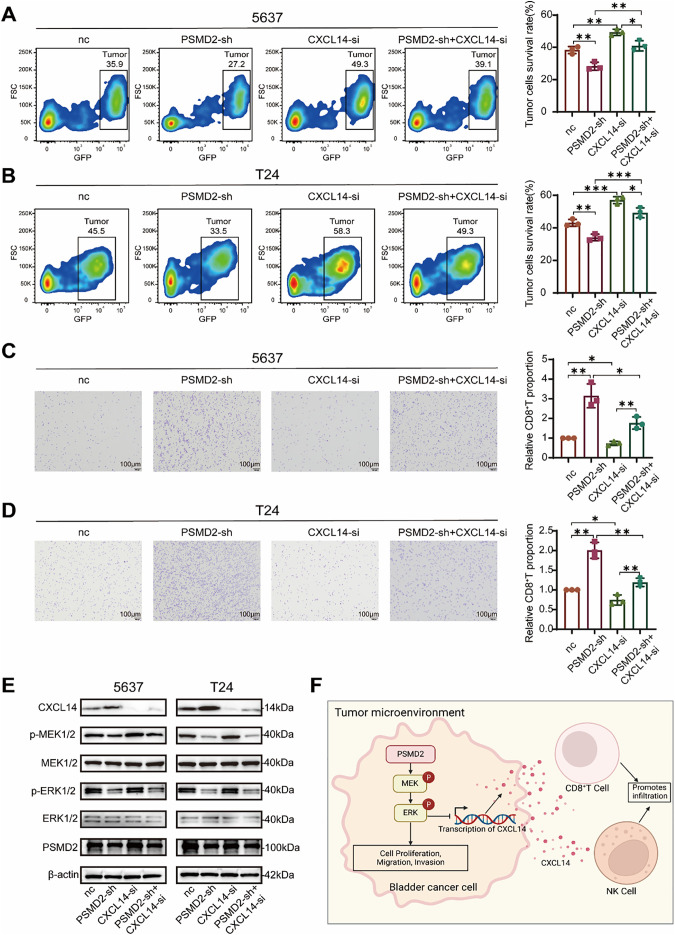
PSMD2 Suppresses Chemokine CXCL14 Expression via the mitogen-activated protein kinase (MAPK) Pathway. (**A**): Flow cytometry analysis the survival rate of the 5637 cells after co-culture with CD8^+^ T cells in the nc group, PSMD2-sh group, CXCL14-si group and PSMD2-sh + CXCL14-si group *in vitro*. *p < 0.05, **p < 0.01. (**B**): Flow cytometry analysis the survival rate of the T24 cells after co-culture with CD8^+^ T cells in the nc group, PSMD2-sh group, CXCL14-si group and PSMD2-sh + CXCL14-si group *in vitro*. *p < 0.05, **p < 0.01, ***p < 0.001. (**C**): Transwell assay was performed to analyze the migratory capability of CD8^+^ T cells in response to the nc group, PSMD2-sh group, CXCL14-si group, and PSMD2-sh + CXCL14-si group of 5637 cells. *p < 0.05, **p < 0.01. Scale bars: 100 μm. (**D**): Transwell assay was performed to analyze the migratory capability of CD8^+^ T cells in response to the nc group, PSMD2-sh group, CXCL14-si group, and PSMD2-sh + CXCL14-si group of T24 cells. *p < 0.05, **p < 0.01. Scale bars: 100 μm. (**E**): Expression of CXCL14 and MAPK pathway proteins (p-MEK, p-ERK, MEK, ERK) in 5637 and T24 cells from the nc, PSMD2-sh, CXCL14-si, and PSMD2-sh + CXCL14-si groups. (**F**): Mechanism of PSMD2 function in BCa

To further validate the functional relationship between PSMD2, immune cells, and chemokines, an *in vitro* co-culture killing assay was performed. Following co-culture with CD8^+^ T cells, the proportion of remaining tumor cells was significantly lower in the PSMD2-sh group compared with the nc group (Fig. S3A), while the CXCL14-si group showed increased residual tumor cells, indicating that CXCL14 deficiency impairs CD8^+^ T cell-mediated cytotoxicity. In rescue experiments, CXCL14 knockdown in PSMD2-deficient cells reversed the enhanced killing effect, leading to an increased proportion of remaining tumor cells compared with the PSMD2-sh group (Fig. 6A,B).

Consistently, transwell migration assays showed that supernatants from PSMD2-sh cells strongly promoted CD8^+^ T cell migration (Fig. S3B), whereas supernatants from the CXCL14-si group exhibited reduced chemotactic ability. In contrast, the CD8^+^ T cell migration level in the rescue group was significantly attenuated compared with that in the PSMD2-sh group (Fig. 6C,D).

WB analysis revealed that PSMD2 knockdown decreased p-MEK/p-ERK levels and upregulated CXCL14 expression (Fig. S3C). CXCL14 silencing alone did not significantly affect MEK/ERK phosphorylation, while the rescue group exhibited phosphorylation levels similar to those in the PSMD2-sh group (Fig. 6E).

In summary, PSMD2 promotes BCa progression by directly activating the MAPK pathway and by suppressing CXCL14 transcription and expression through this pathway, thereby remodeling the immune microenvironment (Refer to Fig. 6F for the schematic diagram).

### 3.7 Performance of RF Models in High vs. Low PSMD2 Expression Prediction Using Tumor and Normal Patch Features

**Figure 7:**
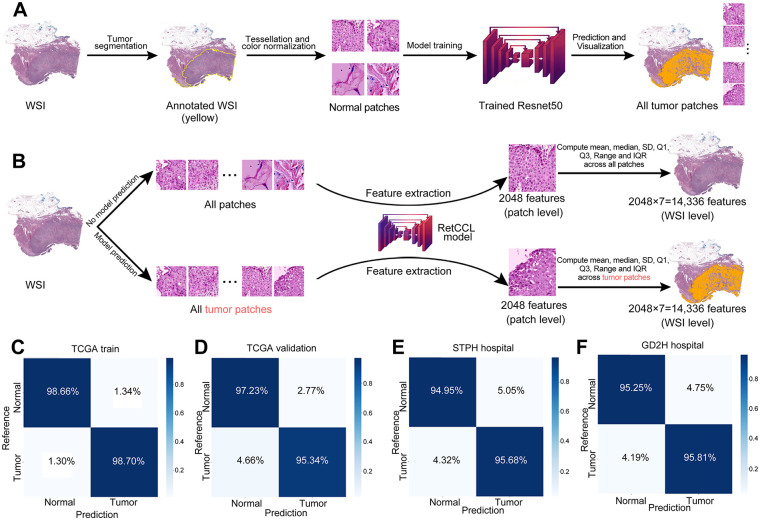

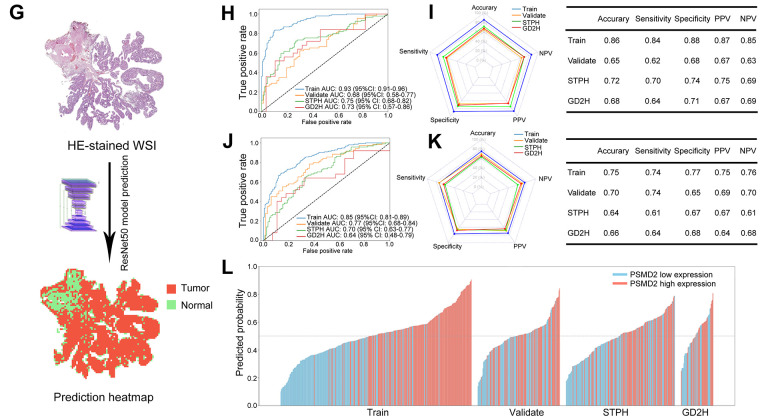
Performance of random forest (RF) models predicting high vs. low PSMD2 expression. (**A**): Transfer learning of the ResNet50 model to identify tumor and normal patches. (**B**): Two methods for extracting whole-slide images (WSI)-level deep learning features. (**C**–**G**): Confusion matrices display the model’s performance in (**C**) TCGA training set, (**D**) TCGA validation set, (**E**) The Shanghai Tenth People’s Hospital (STPH) external validation set, and (**F**) Guangdong Second Provincial General Hospital (GD2H) external validation set. (**G**) Representative example of a hematoxylin and eosin (H&E) stained whole-slide images (HE-stained WSI) with the ResNet50 model’s tumor and normal predictions shown as a heatmap overlay. (**H**): Receiver Operating Characteristic (ROC) curves for the RF model using tumor and normal features. (**I**): Radar plot and table summarizing accuracy, sensitivity, specificity, positive predictive value (PPV), and negative predictive value (NPV) for RF model using tumor and normal features across datasets. (**J**): ROC curves for the RF model using tumor features. (**K**): Radar plot and table summarizing accuracy, sensitivity, specificity, PPV, and NPV for RF model using tumor features across datasets. (**L**): Waterfall plot of predicted probabilities for high and low PSMD2 expression by the RF model using tumor and normal features

To explore the correlation between PSMD2 expression and pathological tissues in patients with BCa, a ResNet50 model was developed to differentiate tumors from normal tissue patches (Fig. 7A,B). Following transfer learning, the ResNet50 model exhibited high accuracy in distinguishing tumor from normal tissue across the TCGA training set (Fig. 7C), the internal validation set (Fig. 7D), and two external validation sets (STPH and GD2H) (Fig. 7E,F). These results demonstrate the model’s robust performance and strong generalizability across both internal and external datasets.

An RF model was developed using deep learning features extracted from tumor and normal tissue patches (Fig. 7G). The RF model showed strong performance in the TCGA training set, the internal validation set, and the two external validation sets (STPH and GD2H) (Fig. 7H,I). While the model performed excellently in the training set, a slight decline in performance was observed in the validation sets, though it still maintained reasonable generalizability. Similarly, an RF model trained only on features from tumor patches exhibited a comparable trend, though it generally performed worse than the model that included both tumor and normal tissue features (Fig. 7J,K). Variability between datasets, especially in the GD2H external validation set, posed challenges for model performance.

The waterfall plot (Fig. 7L) illustrates the model’s ability to effectively distinguish PSMD2 expression levels in the training set, although its discriminative ability was somewhat reduced in the validation sets. Overall, incorporating normal tissue features improved the predictive capability and stability of the model. Both RF models successfully predicted PSMD2 expression levels based on pathological features, with the combined tumor-normal model showing superior predictive performance.

### 3.8 Discovery of Small-Molecule PSMD2 Inhibitors through Virtual Screening and Structural Analysis

**Figure 8:**
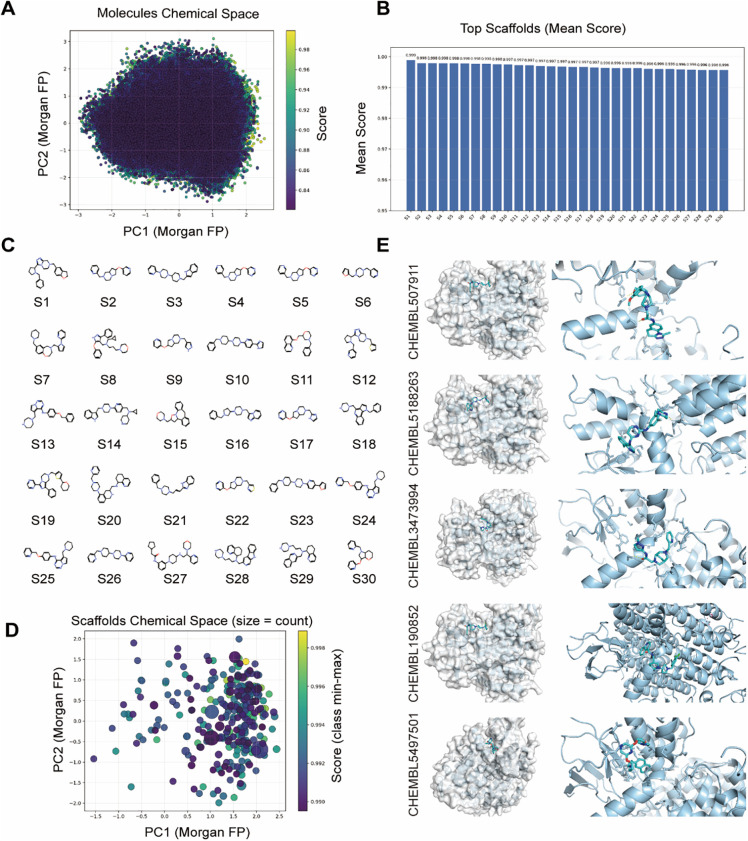
Discovery of Small-Molecule PSMD2 Inhibitors through Virtual Screening and Structural Analysis. (**A**): Principal component analysis (PCA) projection of the top 100,000 compounds in two-dimensional chemical space. (**B**): Murcko scaffold analysis showing score distributions of 30 structural classes. (**C**): Chemical structures of 30 representative Murcko scaffold classes. (**D**): Tanimoto similarity-based Butina clustering of the top 100,000 compounds, visualizing the top 300 clusters. (**E**): Molecular docking showing binding modes of the top five scoring compounds (CHEMBL507911, CHEMBL5188263, CHEMBL3473994, CHEMBL190852, CHEMBL5497501)

These findings highlight the critical role of PSMD2 in BCa progression, suggesting that targeting PSMD2 could be a promising therapeutic strategy. To validate this hypothesis in a preclinical setting, specific PSMD2 inhibitors were identified through rapid virtual screening of the ChEMBL [27] compound library using the TransformerCPI2.0 deep learning model. Principal component analysis (PCA) was applied to project the top 100,000 compounds into a two-dimensional chemical space, providing an overview of their molecular distribution (Fig. 8A). Murcko scaffold analysis identified 30 representative structural classes, displaying their score distributions (Fig. 8B) and chemical structures (Fig. 8C), thus revealing preliminary structure-activity relationships. To assess structural diversity, Tanimoto similarity-based Butina clustering was performed on the top 100,000 compounds, with the resulting top 300 clusters visualized (Fig. 8D). Molecular docking of these clusters identified five top-scoring compounds (CHEMBL507911, CHEMBL5188263, CHEMBL3473994, CHEMBL190852, and CHEMBL5497501), with their binding modes shown in Fig. 8E. These integrated virtual screening, scaffold analysis, and docking results successfully identified several promising lead compounds, providing a strong foundation for subsequent experimental validation and drug development.

## 4 Discussion

BCa is among the most prevalent urological cancers worldwide and ranks as the 9th leading cause of cancer-related mortality globally [1]. Due to its high propensity for progression and recurrence, BCa imposes significant health and economic burdens on society [28]. Tumor progression is closely linked to both the intrinsic properties of the tumor and its TME. The TME, a complex system comprising tumor cells, surrounding cells, stroma, and blood vessels, plays a pivotal role in tumor progression and the initiation of immune responses [29–32]. This study identified PSMD2 as a key gene involved in BCa progression and immune cell regulation within the TME. PSMD2 directly promotes BCa malignancy through the MAPK pathway while simultaneously reshaping the immune microenvironment.

Extensive evidence suggests that PSMD2 acts as an oncogene in various cancers [6,33]. Detailed studies of its role in cancer progression have revealed that PSMD2, a critical proteasome component, regulates tumor cell apoptosis and proliferation by inducing the ubiquitination and degradation of target proteins [5]. Prior research has demonstrated that PSMD2 knockout reduces proteasome activity, disrupting the balance between phosphorylated AKT and p38, while inducing p21 and leading to growth inhibition and apoptosis in lung cancer cell lines [34]. In addition to its role in proteasome function, PSMD2 activates the mTOR pathway by upregulating ASS1 and inhibiting autophagy [33].

The present study revealed significant upregulation of PSMD2 expression in BCa. PSMD2 knockdown led to a marked reduction in BCa cell proliferation, invasion, and migration, accompanied by a substantial increase in CXCL14 expression. Both *in vivo* and *in vitro* experiments demonstrated that PSMD2 knockdown downregulated MEK and ERK phosphorylation in the MAPK pathway, significantly suppressing BCa proliferation, invasion, and migration while markedly enhancing CXCL14 expression. Co-immunoprecipitation (Co-IP) experiments revealed no direct binding between PSMD2 and MEK/ERK (Fig. S3D), suggesting that PSMD2 may not exert its effects through direct interaction with MEK/ERK. To further explore the relationship among these molecules, screening of the BioGRID [35] database indicated potential interactions between PSMD2, MEK, ERK, and members of the DUSP family. For example, PSMD2 binds to DUSP3 and DUSP15, MEK associates with DUSP19, and ERK interacts with DUSP1, 2, 3, 4, 6, 7, 9, 12, and 19 [36,37] (Supplementary material Table). The DUSP family of proteins dephosphorylates MAPK components (primarily ERK), thereby inhibiting their enzymatic activity. Based on these findings, it is hypothesized that PSMD2 binds to DUSP family proteins and promotes their ubiquitination and degradation, thereby relieving the dephosphorylation constraint on the MAPK pathway and sustaining its activation. Ultimately, this mechanism would ultimately drive bladder tumor progression while suppressing immune responses. However, this hypothesis requires further experimental validation.

CXCL14, a multifunctional cytokine in the TME [38], is a direct predictor of patient prognosis. Reduced CXCL14 expression has been observed in melanoma, colorectal cancer, and hepatocellular carcinoma, correlating with decreased survival rates and shorter patient lifespans [39,40]. The mechanisms underlying CXCL14 suppression vary across cancers, with hypermethylation of its promoter region identified as the dominant mechanism in gastric, colon, and lung adenocarcinomas [41–43]. In HPV-associated tumors, such as head and cervical cancers, downregulation of CXCL14 is linked to the interaction of HPV’s E7 protein with DNMT1, leading to CpG island methylation in the CXCL14 promoter [44,45]. Research by Maehata et al. demonstrated that epidermal growth factor (EGF) binding to its receptor (EGFR) activates the ERK signaling pathway, which results in decreased CXCL14 expression [46]. In the present study, the downregulation of CXCL14 in BCa was found to occur via the MAPK pathway, consistent with findings by Kondo et al. in *Oncogenesis*, which also emphasize the interaction between the MAPK pathway and CXCL14 [47].

In addition to its direct effects, CXCL14 influences tumor progression by interacting with immune cells. Higher CXCL14 levels have been shown to correlate with increased T cell presence in primary tumors and associated lymph nodes [45]. CXCL14 also promotes T cell activation and upregulates MHC class I expression on tumor cells [48], which subsequently enhances responses from NK cells, CD4^+^ T cells, and CD8^+^ T cells within tumor-draining lymph nodes [49]. Consistent with these findings, our study demonstrated a significant upregulation of CXCL14 expression in PSMD2-knockdown cells, which led to increased infiltration of CD8^+^ T and NK cells, thereby inhibiting BCa progression.

In recent years, pathomics has made significant progress in extracting image features associated with BCa tumors. This study demonstrates that incorporating deep learning features from both tumor and normal tissue patches greatly improves the predictive performance of the RF model for PSMD2 expression in patients with BLCA. The combined feature model outperformed the tumor-only model, achieving higher accuracy, sensitivity, specificity, and an AUC of 0.93, with 85.99% accuracy in the TCGA training set and strong performance in both internal and external validation, particularly within the STPH cohort. These results uncover the value of integrating normal tissue features, as they provide a broader tissue context and enhance model generalizability across datasets with varying characteristics and imaging protocols. This approach holds potential clinical utility by leveraging normal tissue signals to differentiate gene expression levels, addressing the challenge of model generalization—a key barrier to translating AI models into clinical practice. The model’s robustness across cohorts highlights the importance of comprehensive tissue context in developing reliable AI diagnostics for pathology, especially in multi-center studies with diverse sample variability.

Limitations of the current study include the relatively small histopathological sample size and the preliminary understanding that PSMD2-enhanced MEK phosphorylation may result from PSMD2-mediated ubiquitination of DUSP protein families, though this mechanism requires further experimental confirmation. Additionally, despite PSMD2’s established role as an oncogenic driver across various cancer types, systematic searches in ChEMBL [27], PubChem [50], BindingDB [51], DrugBank [52], and ZINC [53] using the keywords “PSMD2”, “PSMD2 inhibitor”, and “PSMD2 antagonist” revealed no reported or validated PSMD2-selective small-molecule inhibitors or therapeutics. Although this study has successfully identified several high-potential lead compounds—including CHEMBL507911, CHEMBL5188263, CHEMBL3473994, CHEMBL190852, and CHEMBL5497501—through TransformerCPI2.0 virtual screening, chemical space and scaffold analysis, structural clustering, and molecular docking validation, the efficacy of these compounds requires further experimental verification.

## 5 Conclusion

This study establishes a link between PSMD2 expression in BCa and CXCL14 expression during tumor progression, both *in vitro* and *in vivo*, and explores its impact on tumor-infiltrating immune cells. The results indicate that PSMD2 plays a critical role in driving the malignant progression of BCa and serves as a promising biomarker and therapeutic target for predicting the prognosis of patients with BCa. Pathomics, proven effective in predicting PSMD2 expression levels, has further validated these findings across a range of internal and external training datasets. Furthermore, we have identified potential small-molecule inhibitors of PSMD2, laying a solid foundation for future drug development.

## Supplementary Materials



## Data Availability

The data that support the findings of this study are available from the Corresponding Author, [Yunfei Xu, Yifan Chen, Yang Yan], upon reasonable request.

## Supplementary Materials

The supplementary material is available online at https://www.techscience.com/doi/10.32604/or.2025.072373/s1.

## Abbreviations

BCa: Bladder cancer
BCA: Bicinchoninic Acid
Co-IP: Co-immunoprecipitation
CCK-8: Cell counting kit-8
CCR5: C-C chemokine receptor type 5
CCL3: C-C motif chemokine ligand 3
CCL4: C-C motif chemokine ligand 4
CCL5: C-C motif chemokine ligand 5
CXCL14: C-X-C motif chemokine ligand 14
CXCR3: C-X-C chemokine receptor type 3
CXCL9: C-X-C motif chemokine ligand 9
CXCL10: C-X-C motif chemokine ligand 10
CXCL11: C-X-C motif chemokine ligand 11
EdU: 5-Ethynyl-2^′^-deoxyuridine
EGF: Epidermal growth factor
EGFR: Epidermal growth factor receptor
ERK: Extracellular signal-regulated kinase
FBS: Fetal bovine serum
GD2H: Guangdong Second Provincial General Hospital
GO: Gene Ontology
IF: Immunofluorescence
IHC: Immunohistochemistry
KEGG: Kyoto encyclopedia of genes and genomes
MAPK: Mitogen-activated protein kinase
MEK: Protein kinase kinase
MIBC: Muscle-invasive BCa
nc: Negative control
NK: Natural killer
NMIBC: Non-muscle-invasive BCa
NPV: Negative predictive value
RF: Random forest
ROC: Receiver operating characteristic
PCA: Principal component analysis
PCNA: Proliferating cell nuclear antigen
PFS: Progression-free survival
PSMD2: Proteasome 26S subunit non-ATPase 2
Psmd2-sh: Psmd2 knockdown
PPV: Positive predictive value
qPCR: Quantitative real time PCR
STPH: The Shanghai Tenth People’s Hospital
SGD: Stochastic gradient descent
TCGA: The Cancer Genome Atlas
TME: Tumor microenvironment
WB: Western blot
WGCNA: Weighted Gene Co-expression Network Analysis
WSI: Whole-slide images
