# Supplementary material for: PSMD2-Mediated MAPK Signaling Promotes Bladder Cancer Development and Immune Microenvironment Remodeling
Source: Oncol Res. 2026 Mar 23;34(4):30. doi: 10.32604/or.2025.072373 (PMC13040311; doi:10.32604/or.2025.072373)
Supplement: Supplementary file 1 [file OncolRes-34-72373-s001.docx]

PSMD2-Mediated MAPK Signaling Promotes Bladder Cancer Development and Immune Microenvironment Remodeling

Shuwen Sun^1,2,3,4,#^, Jingcheng Zhang^1,2,3,#^, Zongtai Zheng^5,#^, Yajuan Hao^1,3^, Tianyuan Xu^1,2,3^, Ji Liu^1,3^, Liang Sun^2^, Aimin Wang^2^, Yadong Guo^1,3^, Shiyu Mao^1,3^, Xu Zhang^6^, Yunfei Xu ^1,3,*^, Yifan Chen^1,2,3*^, Yang Yan ^1,2,3*^

^1^Department of Urology, Shanghai Tenth People’s Hospital, School of Medicine, Tongji University, Shanghai 200072, China.

^2^Department of Urology, Bengbu First People's Hospital, Bengbu 233000, Anhui, China

^3^Urologic Cancer Institute, School of Medicine, Tongji University, Shanghai 200072, China.

^4^Department of Nuclear Medicine, Shanghai Tenth People’s Hospital, School of Medicine, Tongji University, Shanghai 200072, China.

^5^Department of Urology, The Affiliated Guangdong Second Provincial General Hospital of Jinan University, Guangzhou 510317, China

^6^Bio-X Institutes (Key Laboratory for the Genetics of Development and Neuropsychiatric Disorders), Shanghai Jiao Tong University, Shanghai 200240, China

*Corresponding Author:

Yunfei Xu: [xuyunfeish@163.com](mailto:xuyunfeish@163.com)

Yifan Chen: chenyifan555@yeah.net

Yang Yan: [yanyang1978@tongji.edu.cn](javascript:void(0))

#These three authors contributed equally to this work.

1 Materials and Methods

1.1 Patients

To build machine learning models for predicting PSMD2 expression using deep learning features from hematoxylin and eosin (H&E) stained whole-slide images (WSIs), we acquired WSIs of BCa patients from TCGA database (https://www.cancer.gov/ccg/research/genome-sequencing/tcga). The selection criteria included: (1) confirmed pathological diagnosis of BCa; (2) availability of RNA sequencing data; (3) comprehensive clinical and pathological data; and (4) high-quality H&E stained WSIs. Exclusion criteria were applied to remove (1) WSIs without evident BLCA lesions; (2) WSIs with substandard scanning quality or those with significant missing values in extracted features; (3) patients lacking RNA sequencing data; and (4) cases where patients received preoperative treatments.

For model validation, we collected additional H&E-stained slides and RNA sequencing data from BLCA patients treated between September 2017 and May 2024 at two external sites: Shanghai Tenth People’s Hospital (STPH) and The Affiliated Guangdong Second Provincial General Hospital of Jinan University (GD2H). The inclusion and exclusion criteria mentioned above were strictly followed. WSIs (in svs format) were scanned at 40× magnification using an automatic digital slide scanner (KF-PRO-120/005, KFBIO Co., Ltd.). In total, 449 WSIs from 378 TCGA patients, 179 WSIs from 163 patients at STPH, and 53 WSIs from 38 patients at GD2H met the criteria and were included in this study. Patient characteristics are outlined in supplementary information Table S2. Since TCGA provided the largest dataset, its WSIs were randomly divided into a training set and an internal validation set with a 7:3 split. WSIs from STPH and GD2H served as external validation sets. The study was approved by the Shanghai Tenth People’s Hospital Ethics Committee (Approval No. 24KT68) and Guangdong Second Provincial General Hospital Ethics Committee (Approval No. 2024-KY-KZ-128-01), and informed consent was collected from all participants.

1.2 Model Construction and Evaluation

To classify tumor and normal patches, transfer learning was implemented using the ResNet50 model in PyTorch (version 2.4.1). The model training ran for 50 epochs with stochastic gradient descent (SGD), an initial learning rate of 0.1, momentum of 0.9, and weight decay set at 0.001. Cross-entropy loss was the chosen criterion, and model performance was assessed on both an internal validation set and two external validation sets from STPH and GD2H.

For distinguishing high and low PSMD2 expression groups, two distinct feature extraction approaches based on deep learning were utilized. In the first approach, all background patches were removed using edge detection, after which the RetCCL model, a variant of ResNet50 optimized for contrastive learning, was used to extract 2048 deep features from the final global average pooling (GAP) layer of both tumor and normal patches. In the second approach, the ResNet50 model trained in this study was applied to isolate tumor patches within the WSIs, and these tumor patches alone were used to derive 2048 features from the RetCCL model’s GAP layer. The RetCCL model, pre-trained on a diverse set of 22,000 WSIs, was chosen due to its enhanced capacity for feature extraction, particularly through its ability to leverage both intra- and inter-class variation in pathology images.

For both methods, the extracted 2048 features from each patch were aggregated at the WSI level by calculating summary statistics such as mean, median, standard deviation (SD), first quartile (Q1), third quartile (Q3), range, and interquartile range (IQR), resulting in a final feature set of 2048 × 7 variables per WSI. Batch correction for the feature sets across different centers was conducted using ComBat to ensure consistency and mitigate batch effects.

Subsequent to feature extraction, Z-score normalization was applied to the training set features, with the derived parameters saved for use on the internal and external validation sets to maintain consistency.

To ensure the robustness and reliability of the developed machine learning models, multiple statistical validation techniques were applied at various stages of the analysis. Initially, for feature selection, Minimum Redundancy Maximum Relevance (mRMR) analysis was employed on the training set to reduce redundancy among features and retain the most informative predictors of PSMD2 expression. This analysis led to the selection of the top 30 features for each feature extraction method, based on their predictive value as determined through mutual information criteria.

Using the features selected by mRMR, random forest (RF) models were constructed on the training set. Models underwent validation on the internal set as well as the two external cohorts.

Hyperparameter tuning was carried out using a grid search strategy, where the number of estimators (ranging from 1 to 200) and the maximum tree depth (ranging from 1 to 15) were optimized to maximize performance. The best model was selected based on the highest average area under the receiver operating characteristic curve (AUC), as AUC is considered a robust metric for evaluating classification performance. In cases where multiple models yielded comparable AUC values, sensitivity and specificity were further evaluated as secondary metrics to ensure that the model performs well across both the high and low PSMD2 expression groups

1.3 Subcutaneous Xenograft Model

All animal studies were approved by the Animal Ethics Committee of STPH (Approval No. SHDSYY-2024-1533). Female SPF-grade BALB/c-nude and C57BL/6J mice (4 weeks old) were purchased from Shanghai Model Organisms and housed in an SPF barrier facility at 20-26°C with 40-70% humidity under a 12-hour light/dark cycle.

A total of 34 mice were used in two independent experiments, comprising 6 groups in total. In Experiment 1 using nude mice, four groups (n=6 per group) were established: two groups were subcutaneously implanted with 5×10⁷ 5637 cells stably expressing either a control shRNA (5637-nc) or a PSMD2-targeting shRNA (5637-shPSMD2), and the other two groups were similarly implanted with T24 cells carrying the control shRNA (T24-nc) or the PSMD2-knockdown construct (T24-shPSMD2). In Experiment 2 using C57BL/6J mice, two groups (n=5 per group) were established: one group was injected with 5×10⁷ MB49 cells expressing a control shRNA (MB49-nc), and the other group received MB49 cells with stable PSMD2 knockdown (MB49-shPsmd2).The sample size was determined based on pilot data showing mean tumor volumes of 1122.16 mm³ (control) versus 369.55 mm³ (experimental), with a pooled standard deviation of 205.12 mm³ (Cohen's d = 3.67). G*Power analysis (two-tailed t-test, α=0.05, power=0.8) indicated a theoretical minimum of n=3 per group to achieve >99% actual power. To ensure robustness and account for biological variability or potential attrition, the sample size was set to a minimum of n=5 per group for the formal experiments. Animals were randomized to groups, and investigators were blinded during tumor measurement and weighing. Tumor length and width were measured weekly, and volume was calculated as V=6π×length×width^2^. Body weight was recorded concurrently. After the 4-week experimental period, mice were euthanized by isoflurane overdose, and tumors were excised and weighed. The primary endpoints were tumor volume and final tumor weight; the secondary endpoint was body weight change. Predefined exclusion criteria included tumor volume exceeding 2000 mm³ or body weight loss >20% (no animals were excluded in this study). Data are presented as mean ± standard deviation. Statistical analyses were performed using GraphPad Prism 8.0, with independent-sample t-tests for between-group comparisons. p-value < 0.05 was considered statistically significant.

1.4 Immunohistochemistry (IHC) and Immunofluorescence (IF)

Fresh tissue specimens are first immersed in a 4% paraformaldehyde solution (Servicebio, G1101, Wuhan, China) for a period of 48 hours. Following fixation, the tissues underwent a standard dehydration process through a graded ethanol series, followed by clearing in xylene and embedding in paraffin blocks. The paraffin-embedded tissues were then sectioned at a thickness of 4 μm. These sections are deparaffinized, rehydrated through a graded series of alcohol, and subjected to antigen retrieval in sodium citrate buffer (Absin, abs9752, Shanghai, China) using a microwave heating method (95-100°C for 15 min), followed by a 20-min cooling period at room temperature(RT). After washing with PBS (Yeasen, 60158ES, Shanghai, China), endogenous peroxidase activity is quenched by incubation in 3% hydrogen peroxide for 15 min. Afterward, they are blocked with 3% BSA (Yeasen, 36101ES) at RTfor 1 hour. The primary antibodies (PSMD2, Ki67 and PCNA, diluted at 1:50) is applied to the tissue sections and incubated at 4 °C for 18 hours. Following this, the sections are incubated with a secondary antibody (HRP-Rabbit, diluted at 1:100) at RT for 30 minutes. Tissue visualization is achieved using Diaminobenzidine for color development, following a 5-minute incubation, and with hematoxylin used as a routine counterstain for nuclei. The sections are then dehydrated and mounted with a neutral resin. The IF closely mirrors the IHC process, with the key distinction being the use of a fluorescently labeled secondary antibody (CY3-conjugated goat anti-Rabbit IgG, diluted at 1:100) to detect the primary antibody (PSMD2, diluted at 1:100). After the secondary antibody incubation, the cell nuclei are stained with 4',6-diamidino-2-phenylindole (Yeasen, 708939ES).(The details of the antibodies are provided in Table S3.)

1.5 Cell Counting Kit‑8 Assay(CCK8)

Seed the cells into each well of a 96-well plate at a density of 1000 cells per well. On days 0 through 5 post-inoculation, add 10 μL of the CCK-8 reagent (Yeasen, 40203ES) to each well and then incubate the plate at 37 °C for 1.5 hours. Measure the absorbance of the samples at a wavelength of 450 nm using a microplate reader (BioTek, Synergy H1, Vermont, USA) to assess cell growth. The calculated result equals the optical density (OD) value of the experimental group minus the OD value of the blank control group.

1.6 Colony Formation Assay

In the colony formation assay, seed 500 cells per well into 12-well plates and incubate for a period of 9 days, until the following termination criteria are met in the control group: colonies are macroscopic, discrete, and approximately circular, each comprising >50 cells, with clear boundaries and no significant overlap. Subsequently, wash the wells three times with PBS, followed by fixation with a 4% paraformaldehyde solution. Apply a 0.5% crystal violet stain (Yeasen, 60506ES) to the cellular colonies for a duration of 20 minutes. Rinse the wells again with PBS and allow the plates to air dry for 30 minutes. Conclude the assay by capturing images of the formed cell colonies using a digital camera (Canon, EOS 750D, Tokyo, Japan). Colonies are quantified using ImageJ software (version 1.53k) with the following criteria applied: only macroscopic, discrete colonies consisting of >50 cells are counted.

1.7 Wound Healing Assay

Cells grown to 95% confluence in a 6-well plate are subjected to a scratch wound. Three parallel scratches are using a 200μL sterile plastic pipette to serve as technical replicates (each approximately 700–800 μm in width). Their migration is tracked by capturing images at the same site with an electron microscope (Nikon, TS2R-FL, Tokyo, Japan) at intervals of 0, 12, and 24 hours. The migratory capacity of the cells is determined by monitoring the reduction in the wounded area over time. Specifically, the scratch area at each time point is measured in standardized pixel units using ImageJ software (version 1.53k). The percentage of wound closure is then calculated using the following formula: Wound Closure (%) = [ (A₀ − Aₜ) / A₀ ] × 100%, where A₀ is the initial scratch area (0 h) and Aₜ is the scratch area at the measurement time point.

1.8 Co-Immunoprecipitation (Co-IP) Assay

For co-immunoprecipitation assays, 5637 and T24 cells were lysed in RIPA buffer (Beyotime, P0013D,Shanghai, China) containing protease inhibitors. Clarified lysates (800 μg of protein) were incubated overnight at 4°C with 5µL specific primary antibodies targeting IgG, PSMD2, MEK, or ERK. Subsequently, 30 µL of Protein A/G agarose beads (Thermo Fisher Scientific, 20421, Massachusetts, USA) were added, and RIPA buffer was supplemented to achieve a total reaction volume of 500 µl. The mixture was then incubated at 4°C for 4 hours with gentle rotation. Bead-bound immunocomplexes were collected by brief centrifugation at 4°C and washed three times with ice-cold PBS to remove non-specific binding. Captured proteins were eluted by boiling in 2× SDS loading buffer for 5 min at 95°C. Eluates were separated by SDS-PAGE and analyzed by western blotting to assess potential interactions between IgG, PSMD2, MEK, and ERK.

1.9 In Silico Virtual Screening and Molecular Docking

The methodological workflow of this study was as follows: First, high-throughput virtual screening was conducted using a pre-trained TransformerCPI2.0 model. The model took the primary sequence of the target protein (input in FASTA format, with only 20 standard amino acids retained and the principal isoform prioritized) and the graph representation of small molecules (SMILES processed via RDKit (version 2025.9.1), including desalting, protonation adjustment at pH 7.4, and structure normalization) as inputs. Through processing by a dual-branch architecture (Transformer for protein encoding and GNN for small molecule encoding), the model output a binding score ranging from 0 to 1. Based on this score, the ChEMBL compound library (version 35) was sorted in descending order to select high-scoring molecules. Next, scaffold-based clustering was performed on the top 100,000 molecules: after cleaning invalid data, RDKit was used to generate and classify Murcko scaffolds, calculate scoring metrics for each class, generate 2048-bit Morgan fingerprints, visualize the chemical space via Principal component analysis (PCA), and perform clustering using the Butina algorithm (abnormal molecules labeled as C-1). Finally, molecular docking was carried out for all molecules in the top 300 clusters: the protein structure (PDB ID: 8QH2) was retrieved from the PDB (https://www.rcsb.org/), preprocessed, and its active pocket was defined using fpocket (version 4.0); small molecules were used to generate 10–50 conformations followed by energy minimization. Docking was performed with AutoDock Vina (version 1.2.6) (exhaustiveness = 32, 5 output conformations), and conformations were sorted by binding affinity scores to complete the screening and validation-related analysis of candidate molecules.

1.10 Statistical Analysis

Quantitative data are presented as mean values ± SEM from at least three independent experiments. All statistical analyses were conducted using GraphPad Prism software (v8.0.2, GraphPad Inc, San Diego, CA, USA). Intergroup differences were evaluated using appropriate statistical tests: two-group comparisons employed unpaired Student’s t-tests, while multiple group comparisons utilized one way ANOVA followed by Tukey’s post-hoc test. A probability value of p < 0.05 was considered statistically significant for all analyses.

**Table S1. Primers**

| **Primer** | **Species** | **Knockdown sequence(5’-3’)** | |
| --- | --- | --- | --- |
| PSMD2 | Homo | F: GCTGGCTCAAATCGTGAAGAT | |
|  |  | R: ATCTTCACGATTTGAGCCAGC | |
| Psmd2 | Mus | F: GGTTATGCCTAGAATCCAA |  |
|  |  | R: TTGGATTCTAGGCATAACC | |

**Table S2. Characteristics of patients with bladder cancer from multiple datasets.**

|  | **Number (%)** | | |
| --- | --- | --- | --- |
| **Characteristic** | **TCGA (n=378)** | **STPH (n=163)** | **GD2H (n=38)** |
| **Sex** |  |  |  |
| **Women, n(%)** | 97 (25.7) | 29 (17.8) | 5 (13.2) |
| **Men, n(%)** | 281 (74.3) | 134 (82.2) | 33 (86.8) |
| **Age (years)** |  |  |  |
| **<65, n(%)** | 137 (36.2) | 49 (30.1) | 18 (47.4) |
| **≥65, n(%)** | 241 (63.8) | 114 (69.9) | 20 (52.6) |
| **Pathologic T stage** |  |  |  |
| **NMIBC (<pT2) , n(%)** | 3 (0.8) | 112 (68.7) | 22 (57.9) |
| **MIBC (≥pT2) , n(%)** | 375 (99.2) | 51 (31.3) | 16 (42.1) |
| **Pathological grade** |  |  |  |
| **Low, n(%)** | 21 (5.6) | 34 (20.9) | 13 (34.2) |
| **High, n(%)** | 354 (93.7) | 129 (79.1) | 25 (65.8) |
| **N/A, n(%)** | 3 (0.8) | 0 (0) | 0 (0) |

Abbreviations: TCGA (The Cancer Genome Atlas), STPH (the Shanghai Tenth People's Hospital), GD2H (Guangdong Second Provincial General Hospital), NMIBC (Non-muscle-Invasive Bladder Cancer), MIBC (Muscle-Invasive Bladder Cancer), N/A (Not Available).

**Table S3. Antibodies**

| **Antibodies** | **Product number** | **Company** | **Province/State** | **Country** |
| --- | --- | --- | --- | --- |
| β-actin | 66009-1-Ig | ProteinTech | Wuhan | China |
| PSMD2 | 14748-1-AP | ProteinTech | Wuhan | China |
| CXCL14 | TD12377 | Abmart | Shanghai | China |
| ERK1/2 | AF0155 | Affinity | Cincinnati | USA |
| MEK1/2 | AF6385 | Affinity | Cincinnati | USA |
| p-ERK1/2 | AF1015 | Affinity | Cincinnati | USA |
| p-MEK1/2 | AF8035 | Affinity | Cincinnati | USA |
| Ki67 | Ki67 | Wanleibio | Shenyang | China |
| PCNA | PCNA | Wanleibio | Shenyang | China |
| HRP-Rabbit | ab6721 | abcam | Cambridge | UK |
| HRP-Mouse | ab6728 | abcam | Cambridge | UK |
| CY3-conjugated goat anti-Rabbit IgG | WLA4136 | Wanleibio | Shenyang | China |


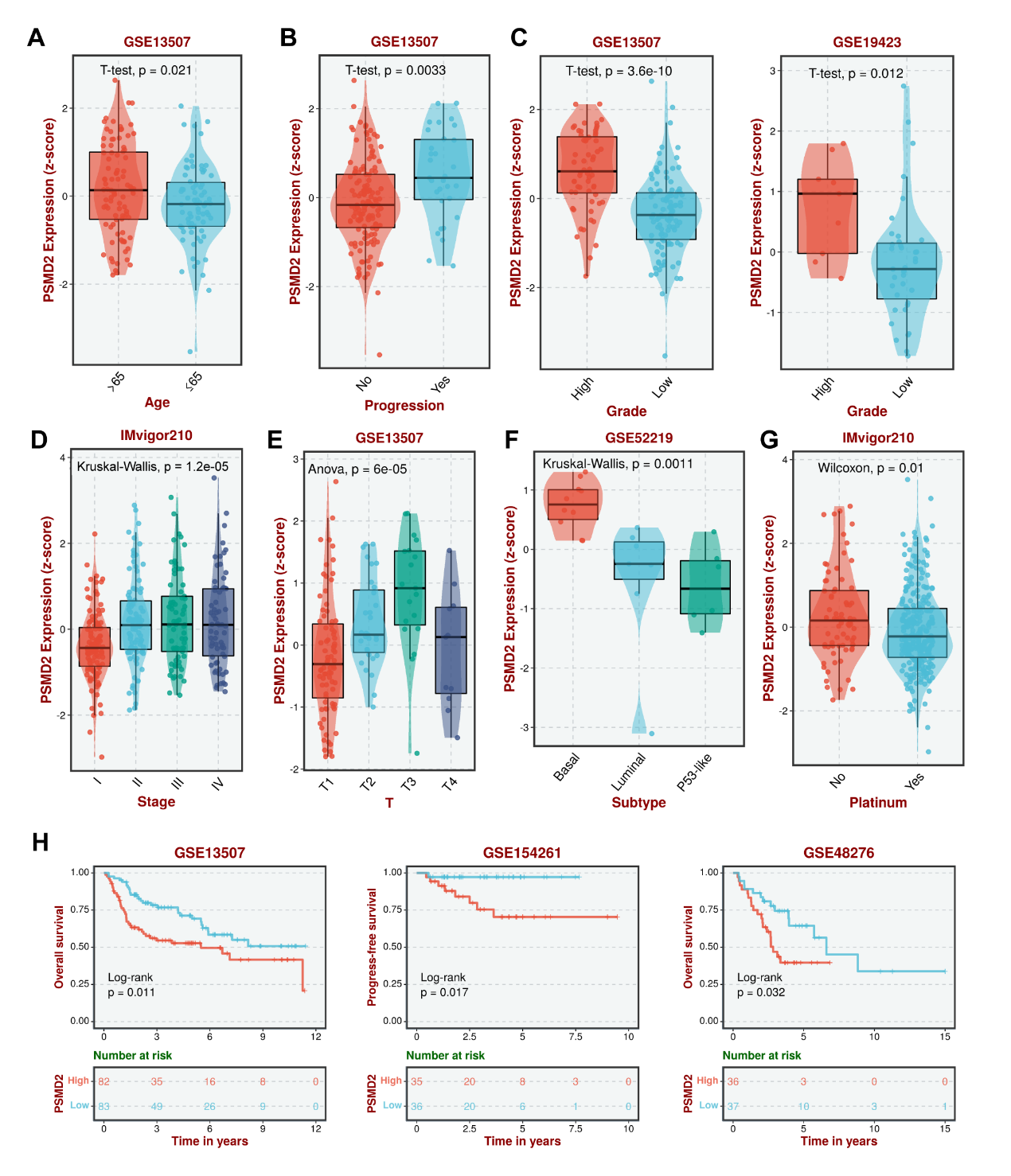


**Figure S1: Multiple datasets analyzed the correlation between Proteasome 26S Subunit Ubiquitin Receptor, Non-ATPase 2 (PSMD2) and the clinical information of bladder cancer (BCa) patients.** (A): The relationship between PSMD2 expression and age. (B): The correlation between PSMD2 expression and progression. (C): Correlation between PSMD2 expression and tumor pathological grade. (D): The correlation between PSMD2 expression and stage. (E): The correlation between PSMD2 expression and T stage. (F): Correlation between PSMD2 expression and molecular typing of BCa. (G): The correlation between the expression of PSMD2 and the efficacy of platinum chemotherapy. (H): The correlation between the expression of PSMD2 and patients' Overall Survival (OS) and Progression-Free Survival (PFS).


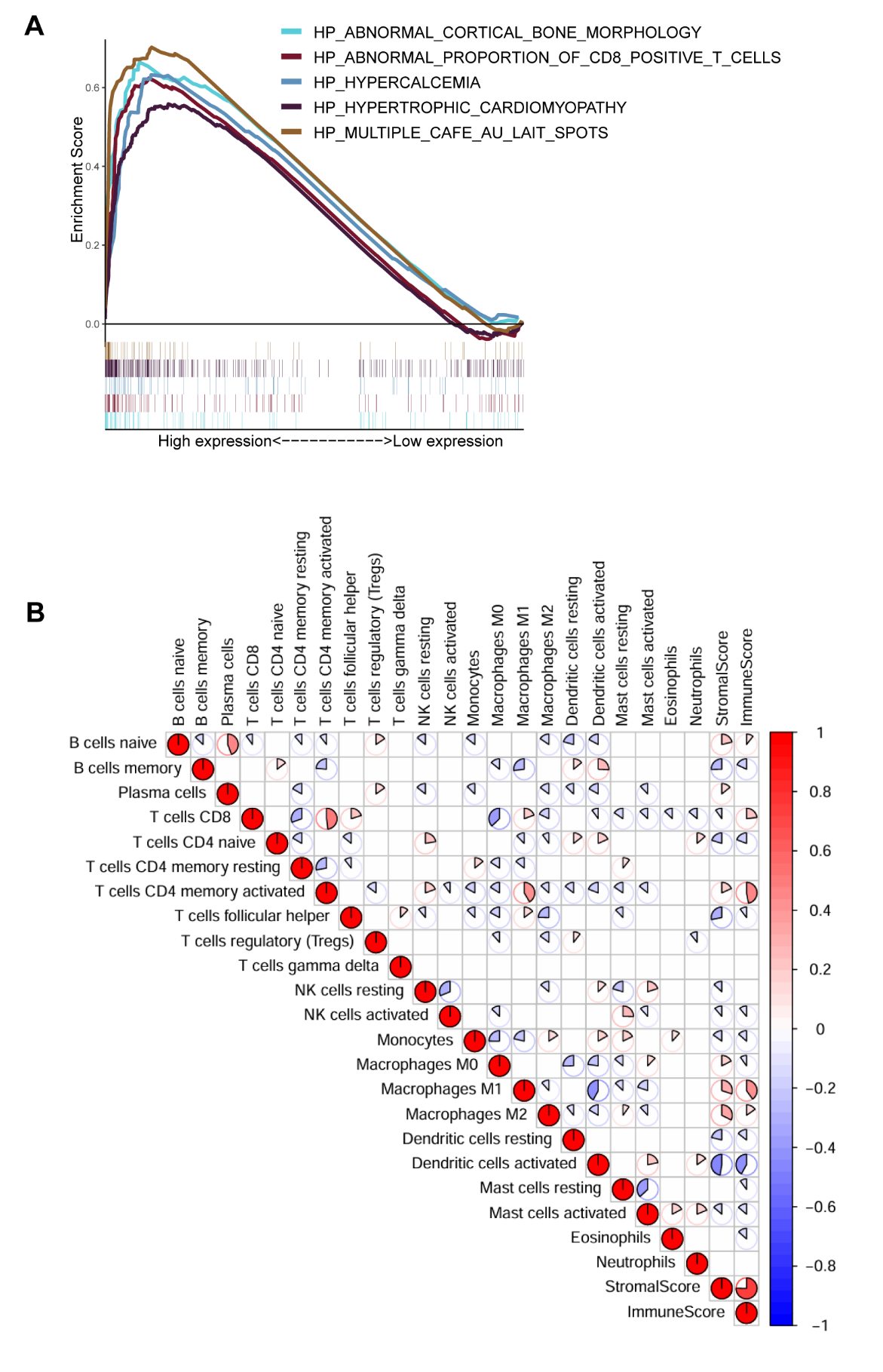


**Figure S2: Biometric analysis revealed that PSMD2 was directly associated with the immune microenvironment of BCa. (A)** Analysis of pathways affected by PSMD2 by Kyoto encyclopedia of genes and genomes (KEGG) enrichment. **(B)** Correlation of PSMD2 with immune score and immune cells.


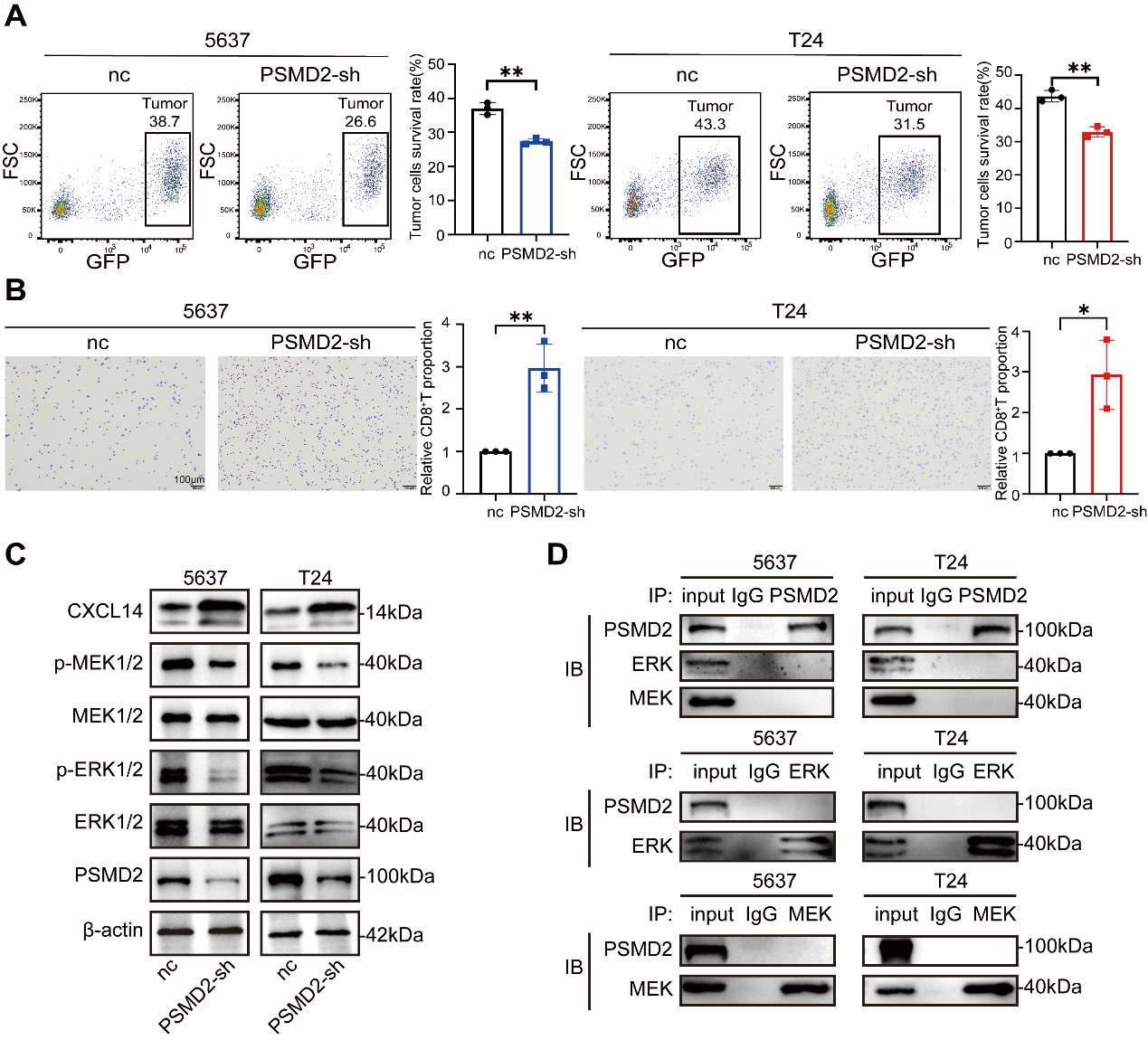


**Figure S3: Effect of PSMD2 on Immune Cell Recruitment and Mitogen-activated protein kinase (MAPK) Signaling. (A)** Flow cytometry analysis the survival rate of the tumor cells after co-culture with CD8+ T cells in the nc group and PSMD2-sh group in vitro. ** p < 0.01. **(B)** Transwell assay was executed to analyze the migratory capabilities of CD8+ T cells following different tumor lines. Scale bars: 100 μm. * p < 0.05, ** p < 0.01. **(C)** Induced expression of CXCL14 and MAPK pathway (mitogen-activated protein kinase kinase (MEK), extracellular signal-regulated kinase (ERK), p-MEK, p-ERK) protein after knockdown of PSMD2 in 5637 and T24 cell lines. **(D)** Co-immunoprecipitation (CO-IP) assays in 5637 and T24 cells using anti-PSMD2, anti-MEK, or anti-ERK antibodies. Input: positive control (total cellular protein); IgG: negative control.
